# Non‐Surgical Hip Augmentation With a PDLLA–Hyaluronic Acid

**DOI:** 10.1111/jocd.70695

**Published:** 2026-03-03

**Authors:** Han Earl Lee, Jovian Wan, Ki Woong Yu, Jong Keun Song, Isabella Rosellini, Sebastien Garson, Hugues Cartier, Benjamin Ascher, Kyu‐Ho Yi

**Affiliations:** ^1^ Opening Plastic Surgery Seoul Republic of Korea; ^2^ Medical Research Inc. Wonju Korea; ^3^ Maylin Clinic (Jamsil) Seoul Korea; ^4^ Pixelab Plastic Surgery Clinic Seoul Korea; ^5^ Avery Beauty Clinic and Avena Aesthetics Indonesia; ^6^ Cabinet Médical Senlis France; ^7^ Centre Médical Saint Jean Arras France; ^8^ SiBUS Inc. Paris France; ^9^ Division in Anatomy and Developmental Biology, Department of Oral Biology, Human Identification Research Institute, BK21 FOUR Project Yonsei University College of Dentistry Seoul Korea

**Keywords:** body contouring, hip augmentation, hyaluronic acid, minimally invasive aesthetics, poly‐D,L‐lactic acid

## Abstract

**Background:**

Hip contouring is experiencing growing demand within aesthetic medicine, a trend particularly pronounced in Asian populations where cultural preferences emphasize harmoniously proportioned feminine silhouettes. While surgical augmentation remains an option, its inherent procedural risks and protracted recovery periods underscore the need for effective minimally invasive alternatives. Volumetric enhancement using biostimulatory agents, such as poly‐D,L‐lactic acid (PDLLA), constitutes a non‐surgical approach.

**Objective:**

This study aimed to evaluate the safety, efficacy, and patient satisfaction of bilateral hip augmentation using PDLLA in a cohort of Korean women.

**Methods:**

A prospective case series was conducted involving 30 Korean women (age range: 24–48 years). Participants underwent bilateral hip augmentation between January and June 2024 using a PDLLA–HA hybrid biostimulator (Juvelook G[export name: Juvelook GLAM]).

The mean total volume administered was 201.5 mL (range: 180–220 mL; approximately 100 mL per side, range: 90–110 mL). Outcomes were assessed using the Global Aesthetic Improvement Scale (GAIS), patient satisfaction scores (0–10 scale), and standardized photography at the 6‐month follow‐up.

**Results:**

All 30 participants completed the study protocol. No significant adverse events were reported. Mean patient satisfaction at 6 months was 8.7/10 (±0.9 SD). Based on GAIS assessments, 27 participants (90%) demonstrated moderate to significant aesthetic improvement. Minor transient complications included swelling (*n* = 13, 43.3%) and mild discomfort (*n* = 8, 26.7%), all resolving spontaneously within 1 week.

**Conclusions:**

PDLLA hip augmentation, using total volumes approximating 200 mL, demonstrates a favorable safety profile and high efficacy in this cohort of Korean women. The results indicate this approach presents a viable minimally invasive alternative to conventional surgical implant procedures.

## 1 Introduction

**FIGURE 1:**
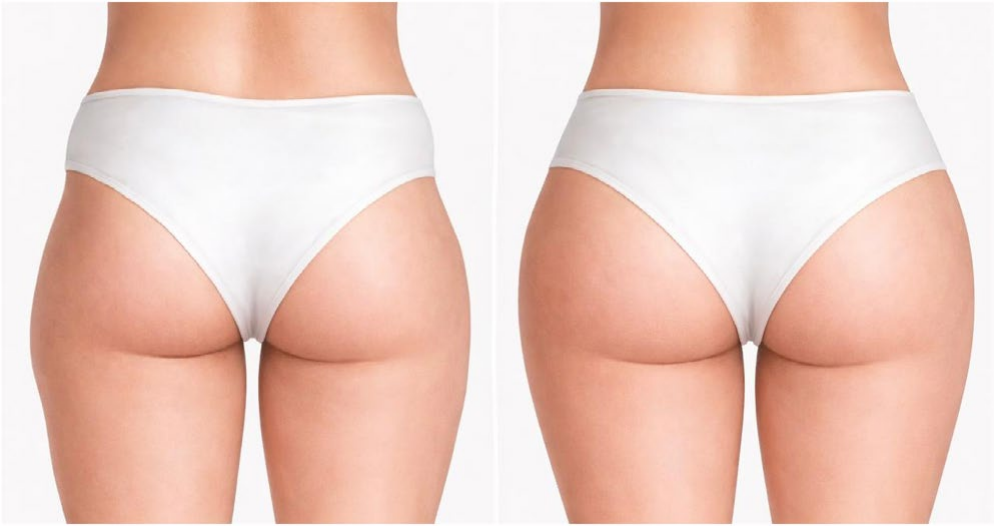
Anatomical comparison of hip contours demonstrating trochanteric depressions. The left panel shows characteristic bilateral concavities located between the iliac crest and greater trochanter, commonly referred to as “hip dips.” The right panel illustrates the desired aesthetic outcome with smooth, continuous lateral hip curves. The ideal uninterrupted contour from the waist to lateral thigh that patients typically seek to achieve through hip augmentation procedures.

The contemporary aesthetic medicine landscape reflects a notable shift toward minimally invasive body contouring, driven by patient demand for safer therapeutic options with diminished morbidity profiles compared to surgical interventions. Within this context, hip augmentation has gained prominence as a procedure addressing the desire for enhanced feminine silhouettes and improved body proportions, particularly targeting the common anatomical feature of trochanteric depressions [1, 2, 3]. These natural concavities, colloquially termed “hip dips,” arise from the anatomical relationship between the iliac crest superiorly, the greater trochanter of the femur inferiorly, and the overlying fascial attachments of the tensor fasciae latae and gluteal muscles. This morphological characteristic manifests as bilateral depressions along the lateral hip contour, often interrupting the desired smooth lateral hip curve (Figure 1) [4].

This trend is particularly evident in Asian populations, where evolving cultural aesthetic ideals increasingly favor continuous curvaceous profiles over traditionally slender physiques. Contemporary Korean aesthetic preferences emphasize smooth, voluptuous hip contours that create an uninterrupted curvaceous silhouette from the waist to thigh [4]. Consequently, anatomical variations resulting in pronounced trochanteric depressions are frequently perceived as aesthetic concerns requiring correction, driving demand for procedures that can create fuller, more rounded hip profiles while maintaining natural proportions.

Traditional hip augmentation methods encompass implants, autologous fat transfer, and injectable fillers. While offering permanent results, implant‐based surgery carries significant risks: infection, capsular contracture, implant migration, and potential revision surgery. Autologous fat transfer, though biocompatible, suffers from unpredictable resorption rates and donor site morbidity. Critically, both surgical approaches necessitate general anesthesia and protracted recovery periods, diminishing their appeal for patients prioritizing minimal downtime [5, 6].

Poly‐D,L‐lactic acid (PDLLA) is increasingly utilized as a biostimulatory agent in non‐surgical contouring. Its amorphous structure, resulting from random 1:1 copolymerization of D‐ and L‐lactic acid monomers, contrasts with the crystalline morphology of poly‐L‐lactic acid (PLLA). This molecular configuration confers a characteristically porous, spherical particle morphology. In vitro and clinical observations suggest this structure may enhance biocompatibility while moderating inflammatory responses relative to some semi‐crystalline polymers. The increased surface area inherent to this morphology potentially facilitates greater collagen neosynthesis, though comparative clinical studies remain limited [7, 8].

Juvelook G (VAIM Inc., Seoul, Korea) represents a recent advancement in PDLLA technology, specifically engineered for body contouring. This formulation combines PDLLA microparticles with a carrier gel, delivering immediate volumization followed by sustained collagen stimulation. Its biphasic mechanism involves initial physical augmentation via particle placement, succeeded by biological enhancement through collagen induction, resulting in durable improvements in tissue volume and quality [9].

Despite PDLLA's increasing use in body contouring, significant evidence gaps persist regarding individualized volume planning for hip augmentation. Existing literature predominantly addresses facial applications or generalized body contouring, with limited focus on the hip region's unique anatomical and aesthetic requirements. Moreover, no studies have specifically evaluated tailored treatment protocols in Korean populations, a demographic prioritizing these procedures.

This case series aims to address these gaps by presenting the first comprehensive analysis of individualized hip augmentation using PDLLA in Korean women. It provides insights into safety, efficacy, and patient satisfaction outcomes associated with tailored volume approaches.

## 2 Materials and Methods

### 2.1 Study Design and Participants

This prospective case series was conducted at our aesthetic clinic in Seoul, Korea, between January 2024 and June 2024. The study included 30 consecutive Korean women seeking hip augmentation who met specific inclusion criteria. All participants provided written informed consent for the procedure and publication of anonymized data.

Inclusion criteria comprised women aged 18–50 years of Korean ethnicity, desire for hip augmentation with realistic expectations, body mass index between 18 kg/m^2^ and 28 kg/m^2^, absence of pregnancy or lactation, no active skin conditions at treatment sites, and ability to attend follow‐up appointments. Exclusion criteria included previous hip surgery or injectable treatments, autoimmune disorders, bleeding diatheses, active infections, unrealistic expectations, and contraindications to local anesthesia.

### 2.2 Pretreatment Assessment

All participants underwent comprehensive pre‐procedural evaluation including detailed medical history, physical examination, and standardized photographic documentation. Photography protocols included posterior and bilateral 45‐degree views under consistent lighting conditions and standardized positioning. Hip morphology was assessed using a modified classification system based on projection and symmetry parameters.

Participants completed pretreatment questionnaires assessing aesthetic concerns, expectations, and baseline satisfaction scores. Body measurements including hip circumference, waist‐to‐hip ratio, and bilateral hip projection were recorded using standardized anatomical landmarks.

### 2.3 Treatment Protocol

#### 2.3.1 Poly‐D,L‐Lactic Acid Preparation

Juvelook G was reconstituted according to the manufacturer's instructions. Each vial was reconstituted with 15 mL of sterile water. Typically 5‐8 vials were used per session, yielding 160‐240 mL total (mean 200 mL ± 28 mL).

#### 2.3.2 Product Composition and Reconstitution

Juvelook G (VAIM Inc., Seoul, Korea) is a lyophilized hybrid biostimulator composed of 170 mg PDLLA and 30 mg non‐crosslinked HA per 200‐mg vial. It is reconstituted with sterile water for injection to yield a homogeneous suspension suitable for subcutaneous deposition.

#### 2.3.3 Volume Planning

Treatment volumes were individualized based on baseline hip anatomy, patient aesthetic goals, body habitus, and desired degree of enhancement. Assessment included measurement of baseline hip projection, waist‐to‐hip ratio, and overall body proportions. The majority of patients received approximately 200 mL total volume, distributed as 100 mL per side, though this was adjusted based on anatomical asymmetries and patient preferences.

#### 2.3.4 Injection Technique

All procedures were performed under strict aseptic conditions with appropriate local anesthesia. The hip region was divided into anatomical zones based on the lateral hip contour, with injection points strategically placed to achieve optimal projection and smoothness.

Local anesthesia comprised 2% lignocaine with 1:200000 adrenaline, injected at entry points and distributed along planned injection tracks. The prepared PDLLA solution was injected using 22‐gauge blunt cannulas via minimal entry points positioned within natural skin creases to minimize visible scarring.

Injection was performed using a fanning technique, distributing the product homogeneously throughout the deep subcutaneous and superficial muscular planes. The individualized volume (ranging from 80 mL–120 mL per side, mean 100 mL ± 14 mL) was delivered systematically across predetermined anatomical zones, ensuring even distribution and optimal contouring. Injection depth was maintained at 15 mm–25 mm to ensure appropriate tissue plane placement while avoiding deeper structures.

#### 2.3.5 Post‐Procedural Care

Participants received standardized post‐treatment instructions including immediate cold compress application for 48 h, avoidance of strenuous activity for 1 week, and gentle massage techniques to promote even product distribution. Follow‐up appointments were scheduled at 6 months post‐treatment.

### 2.4 Outcome Assessment

#### 2.4.1 Primary Outcomes

Aesthetic improvement was assessed using the validated Global Aesthetic Improvement Scale (GAIS), evaluated independently by two plastic surgeons at 6‐month follow‐up visits. The GAIS employs a 5‐point scale ranging from 1 (much improved) to 5 (worse), providing a standardized assessment of treatment outcomes.

Clinical volume maintenance was evaluated through comparative photographic analysis and physical examination, assessing the persistence of hip projection and contour enhancement compared to baseline measurements.

#### 2.4.2 Secondary Outcomes

Patient satisfaction was measured using a 10‐point Likert scale (1 = very dissatisfied, 10 = extremely satisfied) at 6‐month follow‐up visit. Safety outcomes included documentation of all adverse events, categorized by severity, duration, and relationship to treatment.

Anthropometric measurements including hip circumference and projection were recorded at 6‐month follow‐up to provide objective assessment of volume maintenance.

## 3 Results

### 3.1 Demographics

All 30 participants completed the study protocol and attended scheduled follow‐up appointments. The mean age was 32.4 ± 6.8 years (range: 24–48 years). Mean body mass index was 21.3 ± 2.1 kg/m^2^. All participants were nulliparous with no previous aesthetic procedures in the hip region.

### 3.2 Treatment Administration

The individualized injection protocol was successfully completed in all participants without procedural complications. The mean procedure time was 35 ± 8 min. The mean total volume administered was 201.5 mL (range: 180–220 mL; approximately 100 mL per side, range: 90–110 mL), with bilateral distribution typically balanced unless anatomical asymmetries required adjustment. All participants tolerated the procedure well under local anesthesia, with no requirement for additional analgesia or sedation.

### 3.3 Aesthetic Outcomes

**FIGURE 2:**
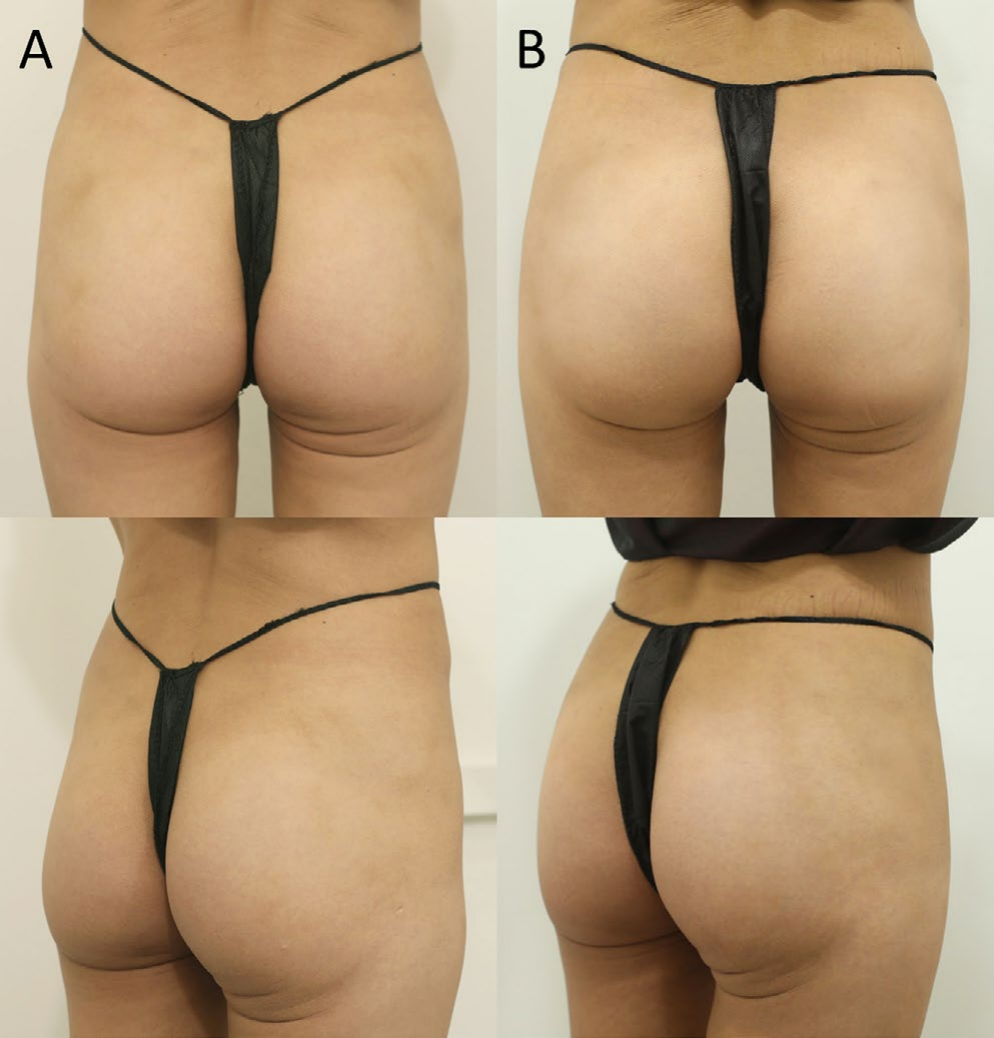
Representative hip augmentation case in a treatment‐naïve 29‐year‐old female. (A) Pretreatment posterior view and 45‐degree posterior view showing baseline hip contour and projection. (B) Six‐month post‐treatment posterior view and 45‐degree posterior view demonstrating enhanced lateral hip curves and improved silhouette following PDLLA injection (total volume 200 mL).

GAIS assessment at 6 months demonstrated excellent results across the cohort. Twenty‐seven participants (90%) achieved moderate to significant aesthetic improvement (GAIS scores 1–2), while the remaining 3 participants (10%) showed mild improvement (GAIS score 3) (Figure 2). No participants demonstrated absence of improvement or worsening outcomes.

Clinical evaluation revealed sustained improvements in hip projection and contour at the 6‐month follow‐up. Standardized photographic analysis confirmed enhanced lateral hip curves and improved waist‐to‐hip ratios across all participants. The treatment consistently produced natural‐appearing augmentation without visible irregularities or asymmetries.

Anthropometric measurements showed significant improvements in hip circumference (mean increase 3.2 ± 0.8 cm at 6 months) and bilateral hip projection (mean increase 1.8 ± 0.4 cm per side at 6 months) compared to baseline values.

### 3.4 Patient Satisfaction

Patient‐reported satisfaction scores remained consistently high at the 6‐month follow‐up. The mean satisfaction score was 8.7 ± 0.9, with twenty‐six participants (86.7%) reporting scores of 8–10, indicating high to extremely high satisfaction levels.

Qualitative feedback revealed particular appreciation for the natural appearance of results, absence of significant downtime, and gradual enhancement that avoided dramatic changes. Participants consistently reported improved confidence and satisfaction with their body image following treatment.

### 3.5 Safety Profile

The treatment demonstrated an excellent safety profile with no serious adverse events reported throughout the study period. All documented adverse events were mild, transient, and self‐limiting.

The most common adverse event was injection site swelling, occurring in 13 participants (43.3%) and typically resolving within 3–5 days with conservative management. Eight participants (26.7%) experienced mild discomfort at injection sites, managed effectively with oral analgesics. Five participants (16.7%) developed minor bruising, which resolved spontaneously within 1 week.

No instances of infection, granuloma formation, product migration, or vascular compromise were observed. All participants maintained normal sensation and function throughout the follow‐up period.

## 4 Discussion

This case series presents an evaluation of individualized hip augmentation using PDLLA (Juvelook G. VAIM Inc., Seoul, Korea) in Korean women. The findings contribute evidence regarding the safety and efficacy of this minimally invasive approach.

The consistently positive outcomes may be attributed to both the biphasic nature of the PDLLA–HA formulation and the individualized injection protocol. PDLLA microparticles induce long‐term collagen formation, while non‐crosslinked HA provides short‐term hydration and early contour support, facilitating immediate correction and improved patient satisfaction.

The synergistic interaction between HA and PDLLA is particularly relevant in high‐volume body contouring procedures. The PDLLA + non–cross‐linked HA formulation does not rely on the high cross‐linker concentrations required in many HA fillers used for gluteal augmentation. This may translate into a lower theoretical risk of delayed inflammatory reactions such as granuloma formation, especially when large filler volumes are required. Beyond volumization, PDLLA‐induced collagen deposition may also reinforce the retinacular cutis and contribute to subtle lifting and tissue‐tightening effects in the gluteal region.

This dual‐phase mechanism may support longer‐term improvement; however, our study evaluated outcomes only up to 6 months, and any durability beyond this period remains hypothetical.

Previous PLLA/PDLLA studies have suggested greater longevity compared with HA fillers due to biostimulatory collagen induction [8], although no direct comparative studies exist for the hip region. Since no commercially available injectable contains pure PDLLA, distinctions between “pure” and hybrid forms are not applicable. Instead, differences noted clinically reflect handling characteristics between PDLLA–CMC and PDLLA–HA formulations.

Although no peer‐reviewed comparative viscosity or integration studies have evaluated PDLLA–CMC versus PDLLA–HA systems, clinicians frequently report smoother handling and easier dispersion with PDLLA–HA hybrids. These impressions derive from procedural experience rather than controlled comparative data and should therefore be interpreted with caution.

The stability observed at 6 months is consistent with the known biostimulatory phase of PDLLA (3–6 months). However, the long‐term durability of PDLLA‐based materials in hip augmentation has not yet been established. While PLLA used in facial volumization is clinically perceived to maintain effects beyond 1 year, such longevity has not been verified through controlled studies and cannot be assumed for larger, biomechanically dynamic regions such as the hips. Therefore, any projection of outcomes lasting 12–24 months should be considered speculative until supported by dedicated long‐term research.

## 5 Limitations

The absence of HA‐only or PDLLA–CMC comparator arms limits direct attribution of volumetric longevity to specific components. Future randomized studies comparing hybrid and monocomponent formulations would help clarify relative contributions to long‐term efficacy. Additionally, the study lacked objective volumetric assessment methods such as MRI or 3D surface scanning, which limits quantitative analysis of contour changes. The single‐center design and homogenous Korean cohort restrict generalizability. Furthermore, the follow‐up period of 6 months is insufficient to evaluate long‐term biostimulatory duration.

## 6 Conclusions

Juvelook G (export name: Juvelook GLAM) demonstrates consistent, natural, and safe augmentation outcomes in the Korean population, integrating early volumization and sustained collagen induction. This hybrid biostimulator may serve as an optimal minimally invasive solution for patients seeking natural hip enhancement without surgical intervention.

## Author Contributions

All authors have reviewed and approved the article for submission.

Conceptualization, Han Earl Lee, Jovian Wan, Ki Woong Yu, Jong Keun Song, Sebastien Garson, Hugues Cartier, Benjamin Ascher, Kyu‐Ho Yi, Isabella Rosellini.

Writing – Original Draft Preparation, Jovian Wan.

Writing – Review and Editing, Han Earl Lee, Jovian Wan.

Visualization, Han Earl Lee, Jovian Wan, Ki Woong Yu, Jong Keun Song, Sebastien Garson, Hugues Cartier, Benjamin Ascher, Kyu‐Ho Yi.

Supervision, Kyu‐Ho Yi.

## Funding

The authors have nothing to report.

## Ethics Statement

This study was conducted in accordance with the ethical principles of the Declaration of Helsinki. As this was a retrospective analysis of anonymized clinical data, formal Institutional Review Board (IRB) approval was not required. All data were handled in a de‐identified manner to ensure patient confidentiality.

## Consent

Informed consent was waived due to the retrospective nature of the study and the use of anonymized data.

## Conflicts of Interest

The authors declare no conflicts of interest.

## Supporting information


**Data S1:** Video Image.

## Data Availability

The data that support the findings of this study are available on request from the corresponding author. The data are not publicly available due to privacy or ethical restrictions.

## References

[1] T. S. Decates , T. Schoonen , A. J. Onderdijk , M. van Leerdam , and P. J. Velthuis , “A Retrospective Study of Nonsurgical Management Options for Permanent Filler Complications of the Gluteal Region,” Plastic and Reconstructive Surgery ‐ Global Open 13, no. 6 (2025): e6815. 40487830 10.1097/GOX.0000000000006815 PMC12144638

[2] L. Lourenco , H. Medeiros , L. Ferreira , N. de Favoretto Dias Oliveira , R. Lopes , and R. Sigrist , “Hip Dips Technique: Filling Lateral Depressions With Hyaluronic Acid of Large Particles,” Skin Health and Disease 4, no. 6 (2024): e461. 39624740 10.1002/ski2.461 PMC11608902

[3] P. Crabai , F. Marchetti , F. Santacatterina , S. Fontenete , and T. Galera , “Nonsurgical Gluteal Volume Correction With Hyaluronic Acid: A Retrospective Study to Assess Long‐Term Safety and Efficacy,” Plastic and Reconstructive Surgery 12, no. 5 (2024): e5792. 38726041 10.1097/GOX.0000000000005792 PMC11081610

[4] R. H. Park , P. L. Myers , and H. N. Langstein , “Beliefs and Trends of Aesthetic Surgery in South Korean Young Adults,” Archives of Plastic Surgery 46, no. 6 (2019): 612–616. 31079443 10.5999/aps.2018.01172 PMC6882691

[5] R. J. Troell , B. Eppley , and S. Javaheri , “Evolving Clinical Experiences in Aesthetic Hip Implant Body Contouring,” Aesthetic Surgery Journal 42, no. 8 (2022): Np516–np530. 35381058 10.1093/asj/sjac064

[6] S. G. Kazmouz , C. A. Riccio , K. Patel , O. Haran , and D. Shifrin , “Implant‐Based Gluteal Augmentation: Comparing Complications Between Single‐ and Double‐Incision Techniques,” Aesthetic Plastic Surgery 48, no. 17 (2024): 3406–3412. 38839612 10.1007/s00266-024-04130-x

[7] G.‐H. Zhang , R.‐X. Hou , D.‐X. Zhan , Y. Cong , Y.‐J. Cheng , and J. Fu , “Fabrication of Hollow Porous PLGA Microspheres for Controlled Protein Release and Promotion of Cell Compatibility,” Chinese Chemical Letters 24, no. 8 (2013): 710–714.

[8] R. Fitzgerald , L. M. Bass , D. J. Goldberg , M. H. Graivier , and Z. P. Lorenc , “Physiochemical Characteristics of Poly‐L‐Lactic Acid (PLLA),” Aesthetic Surgery Journal 38, no. 1 (2018): S13–S17. 29897517 10.1093/asj/sjy012

[9] J. Wan , S. B. Seo , S. E. Yoon , and K. H. Yi , “The Efficacy of Combined Microneedling and Topical Poly‐d,l‐Lactic Acid (Juvelook G) Application for Facial Pore Reduction and Skin Texture Improvement,” Plastic and Reconstructive Surgery 13, no. 6 (2025): e6838. 40469551 10.1097/GOX.0000000000006838 PMC12136670

