# Supplementary material for: Non‐Surgical Hip Augmentation With a PDLLA–Hyaluronic Acid
Source: J Cosmet Dermatol. 2026 Mar 3;25(3):e70695. doi: 10.1111/jocd.70695 (PMC12957896; doi:10.1111/jocd.70695)
Supplement: Supplementary file 1 — Data S1: Video Image. [file JOCD-25-e70695-s001.pdf]

Transcription for Embedded Video (Video S1)

Note: No audible narration was detected in the provided video file; this transcript is a visual description of on-screen actions.

00:00–01:45 Pre-procedure assessment and marking (standing).
• The participant stands while a clinician visually inspects and palpates the gluteal/hip region.
• Treatment zones are outlined on the skin using a red marker. The clinician re-checks the marked area from different angles and adjusts the markings as needed.

01:46–02:20 Positioning and preparation (procedure room).
• The participant is positioned prone on the procedure table.
• The skin is cleansed with antiseptic solution and the field is arranged with sterile drapes.

02:21–03:10 Entry-site preparation.
• Small access points are prepared (wiped/cleansed) and initial injections are administered with a syringe to the planned entry areas.

03:11–08:20 Injection/cannula technique and contouring.
• A longer needle/cannula is introduced through the small entry points and advanced beneath the skin.
• The injectate/intervention material is delivered in multiple passes, with intermittent wiping of the entry sites.
• Multiple syringes are used sequentially. Between passes, the clinician palpates and manually molds the treated area to distribute the material and assess contour/symmetry.

08:21–09:13 Completion and dressing.
• The clinician performs a final visual and tactile assessment.
• Entry sites are cleaned and covered with small gauze pads and transparent dressings.
• A compression/elastic wrap is applied around the upper thigh/hip region. The video ends after the final dressing is secured.
